# Supplementary material for: Clinical Validation and Post-Implementation Performance Monitoring of a Neural Network-Assisted Approach for Detecting Chronic Lymphocytic Leukemia Minimal Residual Disease by Flow Cytometry
Source: Cancers (Basel). 2025 May 17;17(10):1688. doi: 10.3390/cancers17101688 (PMC12110696; doi:10.3390/cancers17101688)
Supplement: Supplementary file 1 [file cancers-17-01688-s001.zip › cancers-3623116-supplementary.pdf]

cancers

MDPI

Supplementary Material

Table S1. Upper verification limits for repeatability and within-laboratory precision obtained from the original CLL MRD method evaluation study.

Sample	Mean	Repeatability SD	Repeatability CV	Within laboratory SD	Within laboratory CV	Upper verification limit - repeatability SD	Upper verification limit - within laboratory SD
BM1	0.81302	0.04137	5.1%	0.09478	11.7%	0.06636	0.16432
BM2	0.36692	0.01196	3.3%	0.02078	5.7%	0.01919	0.03510
BM3	0.36207	0.01705	4.7%	0.01705	4.7%	0.02735	0.02528
BM4	0.53040	0.02882	5.4%	0.04238	8.0%	0.04622	0.06913
BM5	0.72533	0.04308	5.9%	0.07207	9.9%	0.06911	0.11944
PB1	0.03609	0.00416	11.5%	0.00416	11.5%	0.00667	0.0061

The verification limits are based on CLSI EP15-A3:2014 guidelines. Values shown for each specimen include measured means, standard deviations (SD), and coefficients of variation (CV) for both repeatability and within-laboratory precision, along with corresponding upper verification limits that must not be exceeded during the DNN verification process. BM = bone marrow sample; PB = peripheral blood sample.

Table S2. Binary classification performance of DNN + 2nd review approach compared to ground truth (manual analysis).

DNN + 2nd review	Grand Truth (Manual analysis)		Predictive Values
	MRD pos	MRD neg	
**MRD pos**	129	6	PPV = 95.6%
**MRD neg**	0	105	NPV = 100%
	Sensitivity = 100%	Specificity = 94.6%	Accuracy = 97.5%

Table S3. Quantitative method comparison between DNN + 2nd review and manual analysis for percentage of clonal abnormal events.

Statistic	Acceptance Criteria	Observation	Pass/Fail
Correlation Coefficient of Deming regression	≥0.95	0.99	Pass
Tolerable slope deviation	±0.05 (0.95-1.05)	0.99	Pass
MRD-positive cases	>30%	129/240 (53.8%)	Pass
MRD-negative cases	>30%	111/240 (46.2%)	Pass

Table S4. The detailed immunophenotypic features of cases missed by DNN-only analysis.

Case	CD5	CD19	CD20	CD22	CD38	CD43	CD45	CD200	light chain (K or L)
1	neg	pos	neg	pos-dim	neg	neg	pos	neg	pos-dim
2	neg	pos	pos-mod	pos-mod	neg	neg	pos	neg	pos-mod
3	neg	pos	pos-mod	pos-mod	neg	neg	pos	neg	pos-mod

4	neg	pos	pos-mod	pos-mod	neg	neg	pos	neg	pos-mod
5	neg	pos	pos-mod	pos-mod	neg	neg	pos	pos	pos-mod
6	pos	pos	pos-mod	pos-mod	pos-partial	pos-partial	pos	neg	pos-mod
7	pos	pos	pos-mod	pos-mod	neg	neg	pos	pos	pos-dim
8	pos	pos	pos-mod	pos-mod	neg	neg	pos	neg	pos-mod
9	pos	pos-dim	pos-mod	pos-mod	pos-partial	pos-partial	pos	neg	pos-mod
10	pos-dim	pos	pos-mod	pos-mod	pos	neg	pos	pos	pos-mod
11	pos-partial	pos-dim	pos-mod	pos-mod	pos-partial	pos-partial	pos	neg	pos-mod
12	pos-partial	pos	pos-mod	pos-mod	neg	neg	pos	pos-partial	pos-mod
13	pos	pos	pos-dim	pos-dim	neg	pos	pos-dim	pos	pos-dim

Neg – negative; pos – positive; mod – moderate; K – kappa; L – lambda.
